# Pediatric chronic graft-versus-host disease-related dry eye disease and the diagnostic association of potential clinical findings

**DOI:** 10.1038/s41598-023-30288-6

**Published:** 2023-03-02

**Authors:** Hitomi Yagi, Eisuke Shimizu, Ryuichiro Yagi, Miki Uchino, Mizuka Kamoi, Kazuki Asai, Kazuo Tsubota, Kazuno Negishi, Yoko Ogawa

**Affiliations:** 1 Department of Ophthalmology, Keio University School of Medicine, 35, Shinanomachi, Shinjuku-Ku, Tokyo, 160-8582 Japan; 2 Department of Epidemiology, Harvard T.H. Chan School of Public Health, Boston, MA USA; 3 Tsubota Laboratory, Inc, Tokyo, Japan

**Keywords:** Diseases, Medical research

## Abstract

Pediatric graft-versus-host-disease (GVHD)-related dry eye disease (DED) is often overlooked due to a lack of subjective symptoms and reliable testing, leading to irreversible corneal damage. To study the clinical findings contributing to the accurate detection of pediatric GVHD-related DED, a retrospective study of pediatric patients treated with hematopoietic stem cell transplantation (HSCT) at Keio University Hospital between 2004 and 2017 was conducted. Association and diagnostic values of ophthalmological findings for DED were analyzed. Twenty-six patients who had no ocular complications before HSCT were included in the study. Eleven (42.3%) patients developed new-onset DED. The cotton thread test showed excellent diagnostic accuracy in detecting DED (area under the receiver operating curve, 0.96; sensitivity, 0.95; specificity, 0.85) with a cut-off of 17 mm, which was higher than the conventional threshold of 10 mm. Additionally, the presence of filamentary keratitis (FK) and pseudomembranous conjunctivitis (PC) were significantly associated with the diagnosis of DED (*p* value, 0.003 and 0.001 for FK and PC, respectively) and displayed good diagnostic performance (sensitivity, 0.46 and 0.54; specificity, 0.97 and 0.97 for FK and PC, respectively). In conclusion, the cotton thread test with a new threshold, the presence of PC and FK, could be helpful for promptly detecting pediatric GVHD-related DED.

## Introduction

Chronic graft-versus-host disease (GVHD) is a severe and common complication of allogeneic hematopoietic stem cell transplantation (HSCT)^1,2^. The pathophysiology of chronic GVHD is thought to involve inflammation, cell-mediated immunity, humoral immunity, and fibrosis^3^. These immunological cascades related to acute and chronic GVHD cause chronic inflammation of ocular surfaces, such as the lacrimal gland, meibomian gland, conjunctiva, and cornea^4–8^, leading to the development of dry eye disease (DED). DED is the most common ocular complication seen in 50–60% of adult patients with chronic GVHD^9,10^, and the new onset of DED after HSCT is a hallmark of chronic ocular GVHD^11^. In most patients, ocular involvement follows systemic GVHD; however, in a minority of patients, it may precede or occur independently of the systemic disease^12^. Therefore, it is necessary to diagnose ocular GVHD accurately.

The recent increase in the number of HSCTs has been more pronounced in pediatric patients^13^, and the long-term survival rate of these pediatric patients has been on the rise^14–16^. The chronic GVHD has become more frequent with the increasing utilization of unrelated donor transplants and peripheral blood transplants^17,18^. Chronic GVHD, including DED, significantly impacts the quality of life^19–22^, and its management is becoming increasingly important. Over time, DED can cause higher-order aberrations (HOA), corneal surface irregularities, predisposition to infectious ulcers, and secondary corneal scarring, which can lead to irreversible corneal damage^23–25^. HOA in common DED results from corneal surface pathologies, but in GVHD-related DED, the HOA is much more disturbed due to lack or reduced membranous mucin^26^ and leads to severe reduction of QOV^23^. Additionally, ulceration or scarring of the ocular surface in childhood is a lifelong problem. Therefore, preventing such conditions through accurate detection and proper management of DED is vital. In most recent definitions, the diagnosis of DED often requires the evaluation of subjective symptoms^27,28^. In addition, International Chronic Ocular GVHD Consensus Group diagnostic criteria^11^ can be used to diagnose ocular GVHD in adult patients and include subjective symptoms. However, the criteria are difficult to apply for pediatric patients because children often rarely complain of subjective symptoms accurately^29,30^. Therefore, it is necessary to find a diagnostic factor for DED that can be applied to the examination of pediatric patients, and that can be used for early detection.

In this study, we retrospectively investigated the characteristics of pediatric ocular chronic GVHD to detect factors useful for accurately detecting the development of new-onset DED after pediatric allogeneic HSCT.

## Results

**Figure 1:**
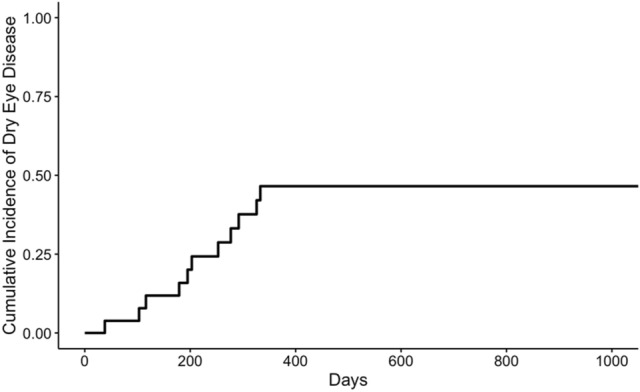
Cumulative incidence of DED in pediatric patients after HSCT. DED was developed within 333 days.

During the study period, 26 patients participated based on the study’s criteria. Characteristics of the 26 patients are listed in Table 1. All patients underwent allogeneic HSCT. Of the 26 patients, 13 (50%) were male, the mean age at HSCT was 8.7 ± 4.6 years, and the median follow-up time was 3.6 (1.3–10.2) years. The most common disease at transplantation was acute lymphoid leukemia. Eleven of 26 patients (42.3%) developed new-onset DED, and there were no differences in age, sex, stem cell source, or the presence of acute or chronic GVHD between patients with DED and those without DED (non-DED patients). Of 11 patients with DED, only 3 (27%) complained of subjective symptoms, including blurring, discharge, and itching. The cumulative incidence of DED after HSCT is shown in Fig. 1. All cases of DED developed within 333 days. Among the patients who developed both systemic GVHD and DED defined as ocular GVHD (*n* = 9), the onset of systemic chronic GVHD was 256. 2 ± 176.5 days and that of ocular GVHD was 220.1 ± 95.9 days (*p* = 0.79). Four patients developed ocular GVHD as an initial sign of systemic GVHD and another two had only ocular GVHD (Supplemental Table 1).

**Table 1:** Characteristics of patients with DED and patients without DED.

	Total	DED	non-DED	*p* value
	(n = 26)	(n = 11)	(n = 15)	
Male, n (%)	13 (50)	5 (45.5)	8 (53.5)	0.64
Mean age at transplantation, y (SD)	8.7 (4.6)	10.2 (4.6)	7.6 (4.1)	0.17
Median follow-up time, y, (IQR)	3.6 (1.3–10.2)	3.7 (1.2–10.6)	3.3 (1.7–9.7)	0.65
Related donor, n (%)	15 (57.7)	5 (45.5)	10 (66.7)	0.26
Acute GVHD ( +), n (%)	18 (69.2)	9 (81.8)	9 (60.0)	0.22
Chronic GVHD ( +), n (%)	16 (61.5)	9 (81.8)	7 (46.7)	0.06
Disease at transplantation, n (%)				
Acute lymphoid leukemia	5 (19.2)	3 (27.2)	2 (13.3)	0.37
Aplastic anemia	4 (15.3)	0 (0)	4 (26.7)	0.06
Juvenile myelomonocytic leukemia	3 (11.5)	0 (0)	3 (20.0)	0.11
Myelodysplastic syndromes	2 (7.7)	0 (0)	2 (13.3)	0.20
Chronic myelogenous leukemia	2 (7.7)	1 (9.0)	1 (6.7)	0.81
Neuroblastoma	2 (7.7)	1 (9.0)	1 (6.7)	0.81
Fanconi anemia	2 (7.7)	2 (18.1)	0 (0)	0.08
Acute myeloid leukemia	2 (7.7)	2 (18.1)	0 (0)	0.08
Other	4 (15.3)	2 (18.1)	2 (13.3)	0.73
Ophthalmic symptoms ( +), n (%)	3 (11.5)	3 (27.2)	0 (0)	0.03

*DED* dry eye disease; *GVHD* graft-versus-host-disease; *SD* standard deviation; *IQR* interquartile range; ( +) positive.

Regarding clinical parameters, patients with DED had significantly shorter cotton thread test (CTT) values (9.0 ± 4.6 mm in patients with DED and 21.0 ± 5.5 mm in patients without DED, respectively; *p* < 0.001) and experienced filamentary keratitis more frequently (45.5% in patients with DED and 0% in patients without DED, respectively; *p* = 0.01) (Table 2). Missing data for Schirmer’s test score affected more than half of the 26 patients; thus, we eliminated Schirmer’s test values as a significant result. Among the patients that developed both filamentary keratitis and DED (n = 5), the mean onset time of filamentary keratitis was 407.2 ± 264.4 days and that of DED was 228.0 ± 264.4 days (*p* = 0.25). Four patients developed filamentary keratitis after DED, and one patient simultaneously developed filamentary keratitis and DED (Supplemental Table 1).

**Table 2:** The association of clinical findings in patients with DED and patients without DED.

	Total (n = 26)	DED (n = 11)	Non-DED (n = 15)	*p* value
Clinical parameters				
Corneal fluorescein staining, points, median (IQR)	1.0 (0.0–3.0)	4.0 (3.0–6.0)	0.0 (0.0–1.0)	< 0.001
Lissamine green, points, median (IQR)	1.0 (0.0–2.0)	2.0 (2.0–3.0)	0.0 (0.0–1.0)	< 0.001
Tear film break-up time, seconds, median (IQR)	6.0 (2.0–10.0)	2.0 (2.0–3.0)	10.0 (6.0–10.0)	< 0.001
Cotton thread test, mm, mean (SD)	15.8 (7.9)	9.0 (4.6)	21.0 (5.5)	< 0.001
Filamentary keratitis (+), n (%)	5 (19.2)	5 (45.5)	0 (0)	0.003
Fibrosis (+), n (%)	6 (23.0)	4 (36.3)	2 (13.3)	0.16
Hyperemia (+), n (%)	4 (15.4)	2 (18.2)	2 (13.3)	0.39
Ocular complications				
Meibomian gland dysfunction, n (%)	9 (34.6)	5 (45.5)	4 (26.7)	0.42
Pseudomembranous conjunctivitis, n (%)	6 (23.0)	6 (54.5)	0 (0)	0.001
Trichiasis, n (%)	4 (15.4)	1 (9.0)	3 (20.0)	0.45
Cataract, n (%)	3 (11.5)	1 (9.0)	2 (13.3)	0.73
Allergic conjunctivitis, n (%)	2 (7.7)	1 (9.0)	1 (6.7)	0.82
Steroid-induced glaucoma, n (%)	2 (7.7)	1 (9.0)	1 (6.7)	0.82
Corneal ulcer, n (%)	1 (3.8)	1 (9.0)	0 (0)	0.23
Corneal neovascularization, n (%)	1 (3.8)	0 (0)	1 (6.7)	0.38
Spontaneous lacrimal punctal occlusion, n (%)	1 (3.8)	0 (0)	1 (6.7)	0.38
Retinal hemorrhage, n (%)	1 (3.8)	0 (0)	1 (6.7)	0.38

*DED* dry eye disease; *IQR* interquartile range; *SD* standard deviation; ( +) positive.

Twenty of 26 patients (76.9%) developed a new onset of ocular complications after HSCT. These included DED (n = 11 [42.3%]), meibomian gland dysfunction (n = 9 [34.6%]), pseudomembranous conjunctivitis (n = 6 [23.0%]), trichiasis (n = 4 [15.4%]), cataracts (n = 3 [11.5%]), allergic conjunctivitis (n = 2 [7.7%]), steroid-induced glaucoma (n = 2 [7.7%]), corneal ulcer (n = 1 [3.8%]), corneal neovascularization (n = 1 [3.8%]), spontaneous lacrimal punctal occlusion (SLPO) (n = 1 [3.8%]) and retinal hemorrhage (n = 1 [3.8%]) (Table 2). Since we follow-up the recipients pre-and post-HSCT, those complications were found to be a new onset of the disease after HSCT. Among the new-onset ocular complications, pseudomembranous conjunctivitis was significantly associated with DED after HSCT (54.5% in patients with DED and 0% in patients without DED; *p* = 0.001). Among the patient that developed both DED and pseudomembranous conjunctivitis (n = 6), the mean onset time of pseudomembranous conjunctivitis was 147.8 ± 103.8 days and that of DED was 224.0 ± 100.1 days (*p* = 0.37) and we found that four patients developed before DED, and two patients developed them simultaneously (Supplemental Table 1). The presence of a pseudomembranous conjunctivitis was found to be associated with higher fluorescein staining scores (5 points, range: 3–8, in DED patients with pseudomembranous conjunctivitis and 3 points, range: 1–4, in DED patients without pseudomembranous conjunctivitis, respectively; *p* ≤ 0.001) and a lower TFBUT (2 points, range: 1–3, in patients with pseudomembranous conjunctivitis and 3 points, range: 2–5, in patients without pseudomembranous conjunctivitis, respectively; *p* = 0.004) among patients with DED.

**Figure 2:**
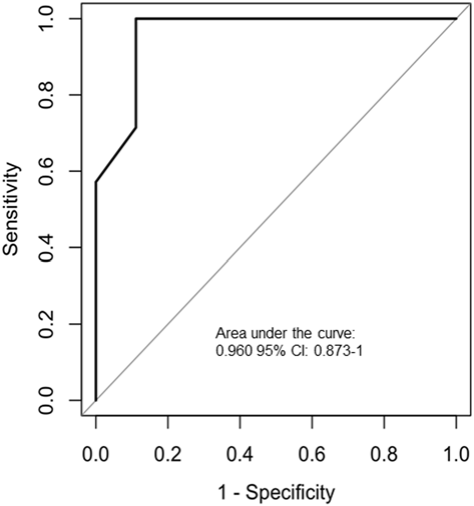
ROC curve of cotton thread test for detecting DED in pediatric patients after HSCT. For the usual cut-off value of 10 mm or less, the sensitivity was 0.71 (95% CI: 0.38–1.00), and the specificity was 0.89 (95% CI: 0.68–1.00), but the cut-off value giving the best balance between sensitivity and specificity was 17 mm with the sensitivity of 0.94 (95% CI: 0.77–1.00), and the specificity of 0.85 (95% CI: 0.63–1.00).

A receiver operating characteristic (ROC) curve was used to assess the CTT discriminating between patients with DED and patients without DED (area under the curve, 0.960 [95% CI: 0.873–1.00]) (Table 3 and Fig. 2). For the usual cut-off value of 10 mm or less, the sensitivity was 0.71 (95% CI: 0.38–1.00), and the specificity was 0.89 (95% CI: 0.68–1.00), but the cut-off value giving the best balance between sensitivity and specificity was 17 mm with the sensitivity of 0.94 (95% CI: 0.77–1.00), and the specificity of 0.85 (95% CI: 0.63–1.00). For filamentary keratitis, the sensitivity was 0.46 (95% CI, 0.18–0.74), and the specificity was 0.97 (95% CI, 0.88–1.00). For pseudomembranous conjunctivitis, the sensitivity was 0.54 (95% CI, 0.26–0.82), and the specificity was 0.97 (95% CI, 0.88–1.00).

**Table 3:** Performance of diagnostic factors for DED related to graft-versus-host disease in pediatric patients.

	Cutoff	Sensitivity (95% CI)	Specificity (95% CI)	+ LR (95% CI)	− LR (95% CI)	Relative risk (95% CI)	AUC (95% CI)
Cotton thread test	≤ 10 mm	0.71 (0.38–1.00)	0.89 (0.68–1.00)	6.43 (0.96–43.23)	0.32 (0.10–1.06)	4.17 (1.15–15.13)	0.96 (0.873–1)
	≤ 17 mm	0.94 (0.77–1.00)	0.85 (0.63–1.00)	6.25 (1.41–27.63)	0.07 (0.01–1.09)	15.00 (1.00–225.3)	
Filamentary keratitis	Positive	0.46 (0.18–0.74)	0.97 (0.88–1.00)	14.67 (0.90–240.38)	0.56 (0.33–0.95)	3.10 (1.56–6.18)	–
Pseudomembranous conjunctivitis	Positive	0.54 (0.26–0.82)	0.97 (0.88–1.00)	17.33 (1.08–278.66)	0.47 (0.25–0.88)	3.55 (1.68–7.48)	–

*DED* dry eye disease; + *LR* positive likelihood ratio; − *LR* negative likelihood ratio; *AUC* area under the receiver operating characteristic curve.

## Discussion

We found that pediatric patients who underwent allogeneic HSCT developed DED most frequently as ocular GVHD, but only 27% complained of their subjective symptoms. Four patients developed ocular GVHD as an initial sign of systemic GVHD, and another two have only ocular GVHD. The CTT showed excellent diagnostic accuracy in detecting DED with a cut-off of 17 mm, which was higher than the conventional threshold of 10 mm. Additionally, filamentary keratitis and pseudomembranous conjunctivitis were significantly associated with the diagnosis of DED and displayed good diagnostic performance.

DED is the most common ocular complication after allogeneic HSCT, with a 50–60% prevalence, consistent with our results with pediatric patients. However, previous reports have shown that the prevalence of DED in children varies from 12.5 to 61.7%^31–40^. This variation in pediatric prevalence may be because diagnostic criteria are not adapted to children. After all, pediatric patients are considered less likely to complain of their symptoms^32,41^. The International Chronic Ocular GVHD Consensus Group diagnostic criteria^11^ can be used to diagnose ocular GVHD in adult patients but include subjective symptoms Ocular Surface Disease Index (OSDI). This is because there is a correlation between adult ocular GVHD-related DED and OSDI^42^. Moreover, several diagnostic criteria for common DED were set for adults, and the presence of symptoms related to DED is mandatory^27,28^. However, pediatric DED could be different from adult DED, especially in their symptoms as shown in pediatric Sjogren’s syndrome^41^. In pediatric Sjogren’s syndrome, of which clinical signs and symptoms of DED are similar to GVHD-related DED, patients do not complain about dry eye symptoms or express symptoms accurately^41^. Therefore, new diagnostic criteria for a pediatric version of Sjogren’s syndrome-related DED are under development^43^. Notably, in our results, only 3 of 11 (27%) patients with DED complained of their newly developed subjective symptoms after HSCT, despite the average age of 8.3 in the situation that we examined almost all patients before HSCT and followed then every 3 to 6 months in our hospital. Considering the youngest case of DED occurred at the age of 2.3, we need to be more aware of objective ocular-surface signs that may be diagnostic factors of DED in pediatric patients after HSCT. Furthermore, four patients developed ocular GVHD as an initial sign of systemic GVHD, and another two have only ocular GVHD. These results may support the importance of diagnosing ocular GVHD and further establishing diagnostic criteria for pediatric ocular GVHD alone.

Our findings demonstrated that the CTT was significantly associated with pediatric chronic GVHD-related DED and had good sensitivity and specificity. In the Japanese dry eye diagnostic and NIH consensus criteria, the scoring form eliminated the CTT and Schirmer’s test because of their low correlation with changes in symptoms^1,44,45^. However, since children rarely complain of subjective symptoms or cannot express them accurately, complementary tests to evaluate tear fluid, such as the CTT, are needed in addition to BUT. Schirmer’s test is difficult for children because it is painful and takes 5 min to complete, and more than half of the patients in our study were unable to take it. Alternatively, the CTT could be helpful to younger children who cannot undergo Schirmer’s test. Despite several studies showing the weak correlation between the CTT and other parameters, including tear meniscus height^46^, TFBUT, and dry eye symptom score by a McMonnies dry eye questionnaire^47,48^, other studies showed that the clinical utility of CTT. They have shown a strong correlation between the value of CTT and Schirmer’s test^49,50^, fluorescein staining, and rose bengal staining^51^, and a moderate correlation with dry eye symptom score by OSDI. CTT is less invasive, takes shorter time (15 s vs. 15 min), and stimulates a lower degree of reflex tearing than Schirmer’s test. Moreover, CTT has been reported to provide reproducible measurements for all ages^52,53^. Therefore, our findings suggested that CTT could be a suitable diagnostic parameter for evaluating tear volume in pediatric patients. It should be noted that the best balance was at a cut-off value of 17 mm rather than the usual cut-off value of 10 mm. Sakamoto R, et al. showed that wet length measured by CTT decreased with age, and significant differences were found for 10 and 19 years and 20 and 29 years (*p* < 0.05) in both the United States and Japan^54^. Thus, a higher cutoff value of 17 mm could be optimal in pediatric patients with higher wet lengths than adults. In line, the CTT should help diagnose the development of DED, and the cut-off value of the CTT may be better to be raised to increase sensitivity in pediatric patients. Nevertheless, pediatric patients often cry out and fail to complete examinations. Therefore, mobile mibography^55^, InflammaDry^56,57^, Smart Eye Camera^58^ would be potential objective and simple measurable tools for DED in the future.

In our study, filamentary keratitis was associated with pediatric GVHD-related DED with very high specificity. Filamentary keratitis consists of a bundle of extracellular DNA (eDNA), a spiderweb-like release of nuclear chromatin complexes by neutrophils^53^. These eDNA webs are defined as neutrophil extracellular traps (NETs), cause inflammation on the ocular surface, and are particularly common in GVHD^59^. These findings are likely due to the low concentration in tear fluid of nucleases (such as DNase I) that can hydrolyze and remove eDNA in patients with GVHD^59,60^. Recent reports have shown that NETs contribute to ocular surface pathological changes such as corneal epitheliopathy, conjunctival scarring, ocular surface inflammation, and meibomian gland disease in patients with GVHD^61^, suggesting that NETs are directly or indirectly involved in the pathogenesis of DED. Though filamentary keratitis is already well known to be more frequently seen in patients with severe DED and often found after the diagnosis of common DED, filamentary keratitis, easily detected by slit lamp biomicroscope, could be a meaningful sign in terms of the pathophysiology of GVHD-related DED and helpful to avoid under-detection during follow-up after HSCT.

We found that pseudomembranous conjunctivitis was significantly associated with the diagnosis of DED and displayed good diagnostic performance. Among six patients who developed both pseudomembranous conjunctivitis and DED, four patients developed pseudomembranous conjunctivitis before the onset of GVHD-related DED, suggesting it could help to predict the onset of GVHD-related DED. In addition, pseudomembranous conjunctivitis was significantly associated with a higher CFS score and lower TFBUT in patients with GVHD-related DED. This finding was consistent with the results from previous studies in the adult population, strongly supporting the validity of our study in the pediatric population. Pseudomembranous conjunctivitis consists of degenerated conjunctival epithelial cells probably attacked by mature T cells in the graft, and macrophages phagocytose these degenerated conjunctival epithelia at an early stage after HSCT, which may lead to the ocular surface being exposed to a cytokine storm. This cytokine storm is likely to promote activating fibroblasts, as reported in pulmonary fibrosis^62,63^, which can cause fibrous obliteration at the lacrimal gland and meibomian gland ducts, resulting in severe DED at a later stage after HSCT^64,65^. We removed pseudomembranous conjunctivitis as soon as we found them, but DED with a high CFS score was observed even after the removal due to rapid fibrosis. The NIH consensus project on chronic GVHD insisted that GVHD-related DED is not just a common DED but a more highly morbid form of DED^66^. GVHD-related pseudomembranous conjunctivitis can cause rapidly progressive dry eye in the pediatric population. Therefore, pseudomembranous conjunctivitis is a significant ocular finding which can determine the prognosis of visual function. Incidentally, two cases of DED and pseudomembranous conjunctivitis occurred at approximately the same time in this study. Uchino M et al. and Hayashi S et al. reported two cases from each study that experienced an overlapping syndrome in which pseudomembranous conjunctivitis occurred almost simultaneously with DED^58,67^. In this case, soluble factors released from pseudomembranous conjunctivitis can stimulate fibroblast chemotaxis at an early stage after HSCT, as reported in pulmonary fibrosis^62,63^ and induce rapidly progressive, severe DED. Although pseudomembranous conjunctivitis would be a predictive finding for the development of severe DED, we should keep in mind that it may indicate the presence of DED simultaneously in patients with overlap syndrome.

There are several limitations to this study. Firstly, we applied the revised 2006 Japanese Diagnostic Criteria for DED by excluding subjective symptoms. However, according to the original criteria, it is defined as probable DED without subjective symptoms. Relatively, the other two elements, tear function abnormality and vital staining, would be more necessary and sufficient to meet the criteria. Since TBUT and Schirmer’s test already were used in dividing the study population into DED and non-DED groups when evaluating the effects of clinical parameters, the grouping error could occur in assessing the impact of TBUT and Schirmer’s test in Table 2. Second, although all the ocular complications occurred after the HSCT, some of them, especially allergic conjunctivitis, might be unrelated to HSCT and caused by other reasons. Third, given its retrospective nature, there were variable intervals between follow-up examinations. Thus, clinical signs such as DED that occurred between visits might have been missed. In addition, the small number of patients could result in too broad a 95% CI for filamentary keratitis and pseudomembranous conjunctivitis to be defined as diagnostic factors. Further study is needed to increase the number of cases.

In conclusion, this is the first retrospective analysis to examine the characteristics of chronic ocular GVHD after pediatric HSCT by focusing on DED and detecting possible clinical hallmarks that help diagnose the development of GVHD-related DED in pediatric patients. Pediatric patients rarely complain of symptoms and cannot be applied to the adult chronic ocular GVHD diagnostic criterion. Therefore, DED after HSCT can often be overlooked in pediatric patients, and DED-type conditions in children can easily be undetected. The CTT and the presence of filamentary keratitis and pseudomembranous conjunctivitis would be helpful for accurately detecting the development of new-onset GVHD-related DED in pediatric patients.

## Methods

### Participants

The ethics committee approved this retrospective study at Keio University School of Medicine (#20,170,350). Informed consents were obtained from all the participants and their guardians through the website at Keio University School of Medicine by posting a detailed written guideline and ethical statement of the present study. This study followed the guidelines of the tenets of the Declaration of Helsinki. Ethical guidelines for clinical research from the Japanese Ministry of Health, Labor, and Welfare indicate the studies which do not involve biological tissue and include reviewing medical records retrospectively; researchers do not need to obtain written informed consent from patients and guardians. Following the guidelines of the ethics committees, we posted a detailed written guideline and ethical statement of the present study, including the background of the study, the purpose of the study, study design, privacy policy, freedom to withdraw, inclusion and exclusion criteria, the factors assessed in the medical records, advantage, and disadvantage of participating the study, disclosure of the data, presenting the data at a conference or in a journal, and contact information.

Since we considered patients equal to or older than 18 as adults, the medical records of consecutive patients less than 18 years of age who received allogeneic HSCT at Keio University Hospital from December 2004 to June 2017 were reviewed retrospectively. Ophthalmic examination for baseline screening is routinely performed before HSCT in our outpatient clinic. All patients underwent standardized clinical and ophthalmological evaluations as described below before HSCT and 3, 6, 9, 12, 18, 24, and 30 months after transplantation. Some patients had additional examinations as indicated according to our follow-up schedule. The inclusion criteria for the study were (1) cases with ophthalmic examinations before HSCT, (2) cases involving no ocular complications before HSCT, and (3) follow-up examinations during at least two years after HSCT. The exclusion criteria for all participants were as follows: (1) a history of previous treatment for ophthalmic diseases and (2) other types of severe DED, including Stevens-Johnson syndrome and ocular cicatricial pemphigoid. (3) Patients who had treatment for other inflammatory diseases, including Sjogren’s syndrome, systemic lupus erythematosus, systemic sclerosis, and juvenile rheumatoid arthritis, that require systemic immunosuppression.

In total, 28 patients met the inclusion criteria, and two patients were excluded according to the exclusion criteria. Twenty-six patients remained in our study, and 11 DED cases and 15 non-DED cases were ultimately included in the primary analysis. The patients were divided into these two groups based on the diagnosis of DED to describe the characteristics of pediatric GVHD-related DED.

### Clinical examination

The ophthalmologists performed ocular examinations at the DED subspecialty outpatient clinic at Keio University Hospital (Y.O., M.U., M.K., and E. S.). The examination included the best corrected visual acuity (BCVA) measurement and anterior and posterior segment examinations. Clinical ocular parameters, based on our DED clinic consensus algorithms, such as the tear-film breakup time (TFBUT), corneal fluorescein staining score (CFS score; 0–9), corneoconjunctival lissamine green staining score (LG; 0–9), degree of fibrosis (0–2), degree of filamentary keratitis (0–2), and degree of hyperemia (0–2), were documented^68^. Schirmer’s test (Sterilized Tear Production Measuring Strips, 4,987,896,590,227; Ayumi Pharmaceutical Corporation, Tokyo, Japan) and the CTT (Zone-Quick Phenol Red Thread Tear Test; 2,564,187, Showa Yakuhin Kako Co., Ltd., Tokyo, Japan) were performed before ocular evaluations to avoid the effect of the fluorescein/lissamine green dye solution. For CTT, a phenol red threat was placed in the lateral lower fornix for 15 s. When the phenol red comes in contact with the tear film, it changes color from yellow to red. The thread was removed after 15 s, and the length of the red portion was measured from the tip regardless of the fold^50^. The tear secretion was measured in both eyes, and the lower value was used for the analysis. Visual acuity was measured using a standard Snellen chart.

### Diagnosis of DED

DED was diagnosed when patients had tear function abnormalities and epithelial damage. We used the Japanese Definition and Diagnosis of Dry Eye 2006^44^ by excluding subjective symptoms. Because pediatric patients rarely present their symptoms accurately or may have no subjective symptoms despite severe ocular signs. Tear function abnormalities included a short TFBUT (≤ 5 s) and the value of Schirmer’s test without anesthesia (≤ 5 mm/5 min). Corneal epithelial damage included positive corneal staining scores (≥ 3 of 9 points) on either fluorescein, Rose Bengal, or LG staining tests. Patients’ subjective ocular symptoms were obtained and written in their medical records during the examinations.

### Statistical analyses

Data were analyzed using R (ver. 3.6.1; The R Foundation for Statistical Computing, Vienna, Austria) and Prism. We compared differences in the occurrence of ocular complications and clinical parameters between patients with DED and patients without DED. The normality of the data set was confirmed using a histogram, the Shapiro–Wilk test, and a quantile–quantile plot. Mann–Whitney *U* test was used when the variables did not have a normal distribution. A two-tailed unpaired *t* test was used when they had equal variance, and Welch’s test was used when the data had an unequal variance for comparison. The values of clinical parameters, including CFS, LG, TFBUT, Schirmer’s test, and the CTT, were obtained during the most severe state. Other clinical parameters, including fibrosis, filamentary keratitis, and hyperemia, were obtained at their appearance. When different values for each eye were obtained, we selected the value of the worst eye. In terms of fibrosis, filamentary keratitis, and hyperemia, a score of 1 or above was considered positive. ROC curves were used to seek the diagnostic accuracy and the cut-off value of the CTT for DED. For the CTT, sensitivity and specificity at a cutoff of 10 mm (as a standard cutoff) and a cutoff determined by the Youden index were calculated. The 95% confidence interval (CI) was generated using 2000 bootstrap samples in the pROC package^69^. The sensitivity and specificity of filamentary keratitis and pseudomembranous conjunctivitis were reported. The Wilcoxon rank test was used to compare differences between DED patients with pseudomembranous conjunctivitis and those without pseudomembranous conjunctivitis to describe the distribution of CFS and TFBUT scores. Continuous variables with normal distributions are presented as the mean ± standard deviation (SD), continuous variables with skewed distributions are shown as the median and interquartile range (IQR), and categorical variables are described as numbers and percentages. *P* values less than 0.05 were considered to indicate statistical significance.

## Supplementary Information

Supplementary Information.

## Data Availability

The datasets generated and analyzed during the current study are available in Supplementary Table 1.
